# Exploratory analysis of traditional risk factors of ischemic heart disease (IHD) among predominantly Malay Malaysian women

**DOI:** 10.1186/s12889-019-6855-5

**Published:** 2019-06-13

**Authors:** Norafidah Abdul Rashid, Azmawati Mohammed Nawi, Shamsuddin Khadijah

**Affiliations:** 1 Vector Borne Disease Control Office, Terengganu State Health Department, Ministry of Health Malaysia, Jalan Kuala Terengganu-Kuala Berang, 21400 Marang, Terengganu Malaysia; 2 Department of Community Health, Faculty of Medicine, Universiti Kebangsaan Malaysia Medical Centre, Jalan Yaacob Latiff, Bandar Tun Razak, 56000 Cheras, Kuala Lumpur, Malaysia

**Keywords:** IHD, Malay, Risk factors, Women, Passive smoking

## Abstract

**Background:**

The risk factors of ischemic heart disease (IHD) specific for women are less well studied. However, knowing the risk factors of IHD for women will empower women themselves to be better informed and thus can help them in decision making concerning their health condition. The objective of this study is to explore the commonly studied risk factors of ischemic heart disease (IHD) among a group of Malaysian women.

**Methods:**

A case control study was conducted among 142 newly diagnosed IHD women patients registered in government hospitals in Terengganu, Malaysia and their 1:1 frequency matched population controls. Data on sociodemographic and socioeconomic profile, co-morbidities, lifestyle factors related to physical activities, dietary fat intake, stress, passive smoking history, anthropometric measurements and biochemical markers were obtained.

**Results:**

Middle aged women were recruited with women diagnosed with diabetes (aOR = 1.92, 95% CI: 1.11–3.31), having low HDL-C (aOR = 3.30, 95% CI: 1.28–8.27), those with positive family history of IHD (aOR = 1.92, 95% CI:1.13–3.26) and passive smokers (aOR = 2.99, 95% CI:1.81–4.94) were at higher odds of IHD.

**Conclusions:**

The findings are useful for public health interventions and policy making focusing on specific women population.

## Background

Ischemic heart disease (IHD) often presents differently in women compared to men. With the increase of obesity in both men and women globally, there is a concern regarding the increase in ischemic heart disease especially among women since obese women are at greater risk of developing IHD [1]. Ischemic heart disease contributed 19.2% of the cause of death in men and 21.2% in women [2]. There are barriers in management of IHD in women, for example women themselves sometimes do not recognize the IHD symptoms [3], and even when they know that they are having heart attack, only 53% will contact the emergency services [4]. Healthcare providers also contribute to lack of care optimization since most are still unaware that there are gender differences in managing IHD as well as their risk factors, for example cigarette smoking [3, 5–7].

The increasing trend of mortality caused by IHD among women can be seen worldwide, but the true picture of IHD among women especially Malay women in Malaysia is unknown due to incomplete registry. In Malaysia, the National Cardiovascular Disease Registry consists of data from hospitals or centers with either resident or visiting cardiologist but the data from primary or even secondary care centers are not included in the registry. A report produced in 2008 from the available registry showed that men were three times more likely than women to have acute coronary syndrome (ACS). However the database also revealed that more women get ACS each year than previously thought with an increasing trend from the third decade of life onwards [8]. In the Malaysian Burden of Disease and Injury study, the prevalence of Disability Adjusted Life Years for ischemic heart disease was 20% among men and 19% among women.

The increasing IHD trend among women may due to the involvement of women in the labour force [9, 10] that leads to occupational hazards [11] such as long working hours, job strain and work-life imbalance. Metabolic syndrome, an important risk factor for cardiovascular disease also shows an increasing trend among women [12]. Urbanization and advanced technology make it easier for women to adopt an unhealthy lifestyle [13]. Common risk factors that contribute to IHD among women based on some previous studies were obesity [14], hypercholesterolemia [15] and diabetes [16].

We hypothesize that more cases of IHD has these traditional factors compared to controls: older age, low educational level and economic status, having professional occupation, consume high fat diet, lack physical activities, have high stress, smoke cigarette, hypertensive, diabetic, obese, high cholesterol, give positive family history of IHD and more likely to be exposed to passive smoking. This study aims to determine which of the traditional or frequently studied factors of ischemic heart disease among Malay women in Malaysia are significantly associated with the disease.

## Methods

### Study design and subjects

A case control study was carried out over a year period (from 1st December 2014 until 30th November 2015) among newly diagnosed IHD female patients registered in government hospitals in Terengganu, a state situated in the east coast of Peninsular Malaysia. Cases were frequency matched (1,1) with controls who were users of the health clinics in the same district where the cases reside. Sample size was calculated via Power and Sample Size 2 program (PS2) and the number of cases needed were between 130 to 150. At the end of the study the number of cases obtained was 142 with the same number for controls.

An IHD case was defined as a patient aged between 30 and 65 diagnosed by medical officers or physicians as having ischemic heart disease and diagnosis included stable/unstable angina, STEMI (ST elevated myocardial infarction) or NSTEMI (Non-ST elevated myocardial infarction), acute coronary syndrome (ACS), ischemic heart disease, arrhythmia/dysrhythmia and congestive cardiac failure (CCF). The age criteria is between 30 to 65 years old, and those above 65 years old were not selected to reduce recall bias. The exclusion criteria were difficult communications due to other medical conditions such as stroke and cases referred from hospitals outside the state to ensure cases and controls were from the same population. Inclusion criteria for controls were those who had no IHD symptoms (for example chest pain or discomfort, fatigue or fainting spells) or were never diagnosed as having IHD by any medical practitioner. Exclusion criteria were those diagnosed as having cancer or any chronic diseases of the kidney, liver, gastrointestinal tract or thyroid as this will alter the controls’ lifestyle factors especially in the diet.

### Study tools and variables definition

Data was collected via face-to-face interviews using Malay language pre-coded questionnaire. However, some parts of the questionnaire were self-administered, while other parts were information on physical examination and treatment received by respondents obtained from their medical records. All interviewers were centrally trained and written informed consent was obtained from all respondents. The questionnaire consists of self-developed items as well as items adapted from other instruments which included The Short Fat Questionnaire, IPAQ-M and GHQ-12.

Traditional risk factors are defined as commonly studied and well known factors that are linked to IHD. These comprise of education level, income, type of occupation, diet intake, physical activity, stress level, smoking, comorbidity, anthropometric measurement, cholesterol level and family history of IHD. Educational level was categorized into low (no formal education or of primary/secondary education) and high (tertiary education e.g. college and universities) education while economic status was defined as the per capita household income in Malaysian Ringgit (RM) for a particular month and based on the Statistical Department Malaysia’s Household Income and Basic Amenities Survey Report 2012, the income was divided into high and low income with the median monthly per capita household income for Terengganu of RM 685 (USD 161) as the cut-off point. Occupation was categorized into non-professional (unemployed and housewives, production operators, service industry, business and agriculture/fishery) and professional (professional occupation in law, education, management and technical expertise).

The diet intake of respondents was measured using the validated Short Fat Questionnaire containing 17 items [17]. The sum of answers to the items were calculated as the total score which can range between 0 to 60 and then categorized into 2 categories as high (scores of more than 27) and low fat intake (scores between 0 and 27). Physical activity information was obtained using the validated IPAQ-M questionnaire [18]. Computation of the total score for the short form of IPAQ-M required summation of the duration (in minutes) and frequency (days) of walking, moderate-intensity and vigorous-intensity activities. It was then categorized into two categories, low (non-active) and high (moderate and high).

Stress was assessed using the validated GHQ-12 questionnaire [19]. From GHQ-12, the mean score of study population was determined and the cut off value was 3.0 with stress defined as mean score of more or equal to 3.0 and no stress is when the mean score was less than 3.0. Smoking variable was categorized into smokers (those who still smoke regardless of number of cigarettes smoked per day) and non-smokers (those who were ex-smokers who had stopped smoking for at least 6 months).

Diabetics are those who were diagnosed by medical doctors, if their venous fasting blood sugar level were more or equal to 7.0 mmol/L or their random blood sugar level of more or equal to 11.1 mmol/L [20]. Hypertension was defined as those who were diagnosed by medical doctors as having persistent elevation of SBP of ≥140 mmHg and/or DBP of ≥90 mmHg [21]. Autoimmune disease was self-reported by respondents (autoimmune diseases such as SLE, rheumatoid arthritis, multiple sclerosis, scleroderma), as diagnosed by physician and positive history of migraine was defined as ever diagnosed as having migraine by medical practitioners, or history of recurrent headaches.

Waist circumference (WC) was measured as the diameter at the level of the midpoint between the iliac crest and the lower border of the tenth rib in cm [22]. Obesity definition was based on the respondents’ body mass index (BMI) with the formula of weight in kilogram over height in meter squared (Wt (kg)/ Ht^2^ (m)). The information was obtained from the medical records and if no such record was found, respondents’ body weight was measured to the nearest 0.1 kg with SECA Digital Flat Weighing Machine. Respondents were weighed barefoot wearing light clothing. Height were measured to the nearest 1 mm with a wall-mounted height board with the following requirements: No shoes, heels together, and heels, buttocks, shoulders and head touching the wall with sight straight forward. BMI values obtained were then categorized into obese (BMI > 30.00 kg/m^2^) and non-obese (BMI ≤ 30.00 kg/m^2^).

The cholesterol levels measured were the LDL-C and HDL-C levels. The information was obtained from patients’ record or if they do not have any recent record of LDL-C level results (within 6 months), they were offered blood test to be done in the health clinics. High cholesterol was when LDL-C level was high and HDL-C level was low [23]. In the analysis, LDL-C level was uses as continuous data while HDL-C was categorized as low (HDL-C level <  1.0 mmol/L) and high (HDL-C level ≥ 1.0 mmol/L).

Positive family history of IHD is when respondents gave positive family history of physician-diagnosed IHD among the first degree relatives. Passive smoking was defined as exposure to tobacco smoke either at home or at workplace.

### Statistical analysis

Data analysis was done using IBM Statistical Package for Social Sciences (SPSS) version 21.0. Bivariate analyses were performed, observing exposure rate for cases and controls and the results of their association tests (Student-t test, Pearson’s chi-squared test) with their respective confidence intervals (95% CI). *P* value of < 0.05 was taken as level of significance. Analysis was then followed by simple logistics regression and results of crude odd ratios and confidence intervals were reported. Next analysis of data used multiple logistic regression and these were done parsimoniously. Using stepwise model building method, variables that were included in the analysis was based on the simple logistics regression analysis where variables that showed significant associations at *p* value less than 0.25 were added as not to miss any of the important variables [24]. For all built models, the models’ goodness of fit was assessed by checking for interactions and multicollinearity and the assumptions tested were Hosmer-Lemeshow test, area under the curve and multivariate outlier. Finally, the final model’s supportive statistics referred to was the Nagelkerke pseudo R^2^, which shows how much the variation in the outcome variable is explained by the logistic model.

## Results

Although Malaysia is a multiethnic country, this was an analysis among predominantly Malay women in Terengganu since in both cases and controls over 98% of the population were Malay women. Terengganu, one of the east coast states of Peninsular Malaysia is in the Malay heartland with over 95% of its population being Malay.

At the end of the study the number of cases obtained was 142 with the same number for controls among women in Terengganu. All cases and controls were comparable in terms of age (52.56 ± 8.65 for cases and 52.27 ± 8.96 for controls), educational level and occupational status. However economic status among cases is higher compared to controls although the relationship is not significant.

Among the traditional factors explored in the crude analysis we found significant associations between IHD and diabetes mellitus (DM), hypertension, waist circumference, HDL-cholesterol level, positive family history of IHD and passive smoking as shown in Table 1.To further explore whether the association between development of IHD in women can be explained by traditional factors, associations using multivariate analysis was done. In this analysis, hypertension and waist circumference were no longer significantly associated with IHD in women (Table 2). Comparing the odd ratios from both simple and multiple logistic regression, the value of four of the variables that were found to be significant namely history of Diabetes Mellitus, HDL-cholesterol level, positive family history of IHD and passive smoker were more or less the same.

**Table 1:** Differences in distribution explored of sociodemographic, socioeconomic, genetic, co-morbidities, anthropometric measurement, biochemical markers, lifestyle and environment factors among Malaysian women

Variables	Cases *N* = 142 (%)	Controls *N* = 142 (%)	Regresion coefficient (b)	Crude *OR*	95% Confidence interval	Wald Stats.	*p* value
I. Sociodemographic & Socioeconomic Factors							
Age (years)^a^	52.56 (8.65)	52.27 (8.96)	0.01	1.004	0.977,1.031	0.08	0.782
Educational level							
Low education	120 (84.5)	115 (81.0)	0.25	1.28	0.69,2.38	0.62	0.433
[High education]	22 (15.5)	27 (19.0)					
Economic Status (Income per capita)							
Low (≤ RM 685)	105 (73.9)	95 (66.9)	0.34	1.40	0.84,2.34	1.68	0.194
[High (> RM 685)]	37 (26.1)	47 (33.1)					
Occupation							
Non- professional	121 (85.2)	119 (83.8)	0.11	1.11	0.59,2.12	0.11	0.743
[Professional]	21 (14.8)	23 (16.2)					
II. Genetic factors							
Family history of IHD							
Yes	60 (42.3)	37 (26.1)	0.73	2.08	1.26,3.43	8.16	**0.004**
[No]	82 (57.7)	105 (73.9)					
III. Co-morbidities, Anthropometric measurement and biochemical markers							
Diabetes Mellitus							
Yes	57 (40.1)	34 (23.9)	0.76	2.13	1.28,3.55	8.41	0.004
[No]	85 (59.9)	108 (76.1)					
Hypertension							
Yes	90 (63.4)	72 (50.7)	0.52	1.69	1.05,2.70	4.63	0.031
[No]	52 (36.6)	70 (49.3)					
Autoimmune Disease							
Yes	4 (2.8)	1 (0.7)	1.41	4.09	0.45,37.03	1.57	0.211
[No]	138 (97.2)	141 (99.3)					
Migraine							
Yes	32 (22.5)	26 (18.3)	0.26	1.30	0.73,2.32	0.78	0.378
[No]	110 (77.5)	116 (81.7)					
Waist circumference^a^	90.1 (14.5)	87.9 (12.7)	0.02	1.02	1.00, 1.04	4.80	**0.029**
Obesity (BMI)							
Obese	43 (30.3)	35 (24.6)	0.28	1.33	0.79,2.24	1.12	0.288
[Non obese]	99 (69.7)	107 (75.4)					
LDL-Cholesterol level (mmol/L)^a^	3.65 (1.38)	3.88 (1.09)	−0.15	0.86	0.71,1.04	2.41	0.121
HDL-Cholesterol level (Category)(mmol/L)							
Low HDL (< 1.0)	21 (14.8)	7 (4.9)	1.26	3.34	1.46,8.57	7.82	**0.005**
[High HDL (≥1.0)]	121 (85.2)	135 (95.1)					
IV. Life style factor							
Dietary Intake (Fat)							
High	31 (21.8)	25 (17.6)	0.27	1.31	0.73,2.35	0.80	0.372
[Low]	111 (78.2)	117 (82.4)					
Physical activity							
Low	39 (27.5)	29 (20.4)	0.39	1.48	0.85,2.56	1.92	0.166
[High]	103 (72.5)	113 (79.6)					
Stress							
Yes	49 (34.5)	42 (29.6)	0.23	1.25	0.76,2.07	0.79	0.374
[No]	93 (65.5)	100 (70.4)					
Smoking							
Yes	3 (2.1)	1 (0.7)	1.11	3.02	0.31;29.20	0.91	0.341
[No]	139 (97.9)	140 (98.6)					
V. Environment factors							
Passive smoking							
Yes	91 (64.1)	53 (37.3)	1.10	3.00	1.85,4.85	19.84	**< 0.001**
[No]	51 (35.9)	89 (62.7)					

^a^mean (± sd); [] reference group

Bold indicate *p*<0.05

**Table 2:** Main traditional factors related to IHD among Malaysian women

Variable	Regression coefficient (B)	*Wald* stat.	*p* value	Adjusted OR	95% CI
Diabetes Mellitus					
Yes	0.651	5.51	0.019	1.92	1.11, 3.31
[No]					
HDL-Cholesterol level (mmol/L) (category)					
Low	1.18	6.10	**0.014**	3.30	1.28,8.27
[High]					
Family history of Ischemic Heart Disease					
Yes	0.65	5.76	**0.016**	1.92	1.13,3.26
[No]					
Passive smoking					
Yes	1.09	18.20	**< 0.001**	2.99	1.81,4.94
[No]					

Forward LR Multiple Logistics Regression model was applied. Nagelkerke R^2^ = 0.182. Multicollinearity and interaction term were checked and not found. Hosmer Lemeshow test (*p* = 0.902), area under the curve (72.1%) were applied to check for model of fitness

[] reference group

Bold indicate *p*<0.05

Women with diabetes mellitus were found to be twice at higher odds of getting IHD (aOR = 1.92. 95% CI: 1.11, 3.31) but the odds of IHD for passive smoking were even higher with women who were passive smokers at 3 times higher odds of getting IHD (aOR = 2.99, 95% CI: 1.81,4.94) compared to those who were not passive smokers and controlling for Diabetes Mellitus, HDL-cholesterol level and positive family history of IHD. In this population of Malaysian women, the adjusted analysis of the traditional risk factors contributed to 18.2% risks of getting IHD.

## Discussion

The increasing trends of Malay ethnicity related more with IHD has been proved by previous comparative study in Malaysia [25]. Therefore, this case control study involved relatively homogenous Malaysian women from the predominantly Muslims and Malay state of Terengganu. Positive family history of IHD and higher risk of IHD in women has been shown in a few studies [26–29]. One study suggested that some IHD risk factors for example hypertension, diabetes mellitus and dyslipidemia tend to aggregate among the family members [26]. The pattern of coronary and ischemic events tends to cluster more in families compared with ischemic stroke [28, 30].

Women diagnosed with DM were twice at higher odds of having IHD compared to controls. It has been found that 60% of rural Malays had comorbidity in their life including DM [31]. Diabetes is more prevalent in women and diabetic women had higher risk of IHD [32–34]. Diabetic women were at higher risk of dying from heart disease compared to men [35] and this is probably attributed to the fact that women had lower HDL-cholesterol level and higher blood pressure than men [36]. Uncontrolled diabetes also has been shown to become one of the common risk factor for cardiovascular disease [37]. One factor that may contribute to this situation is the Malaysian food habit of consuming sweetened condensed milk [31].

Women with low level of HDL-cholesterol increase their odds of having IHD. Studies done by Frikke-Schmidt [38] and Lee et al. [39] showed that low plasma level of HDL-cholesterol increases risks of IHD and it has shown that HDL-C is a stronger predictor of IHD in women as compared to LDL-cholesterol level [40]. There are theories that HDL particle harbor anti-atherogenic effects [38]. However the most common pattern of dyslipidemia among the Asian population was low HDL-C levels within two thirds of them occurring in the absence of any other lipid abnormalities [41] including Malaysia [31].

Our study found lifestyle factors were not statistically significant factors that increase risk of IHD. Dietary fat intake and physical activity for both cases and controls are similar. This might be due to the same food exposure for our study population. The Malays or the so called the *Bumiputra* of Malaysia were rather homogenous in terms of the type of food taken [42]. The Malays are almost all Muslims and in the Malay heartland have very clear gender norms and expectations with regards to lifestyle etc. It was found that women in predominantly Muslim countries are at risk of being physically inactive and this is due to certain religious views stating that it is inappropriate for women to be highly active [43]. In general, women are expected not to smoke and this was seen in the study where we had only 3 and 1 in the cases and controls were smokers respectively.

Passive smoking is an important factor in this population. Those who reported exposure to tobacco smoke had t higher odds of developing IHD compared to those who did not report such exposure. This finding is similar to those of studies elsewhere [33, 44–46]. From a national survey, the National Health and Morbidity Survey (NHMSIII) conducted in 2006, smoking among men was found to be higher among the Malays with a prevalence of 55.9% [47]. The same study also showed that those with lower economic status are more likely to smoke and 70.4% of our study population, both cases and controls were from low socioeconomic background. Thus it is safe to say, that women from our study population has higher chance of being passive smokers. Based on a review of cohort studies, it was also found that compared to male smokers, women who smoke have a 25% greater relative risk of coronary heart disease, independent of other IHD risk factors [41].

Although our study has very few women smokers, those women were surrounded by men who smoke. Among cases, more than 60 and 37.2% of controls were exposed to tobacco smoke. These women were exposed to second hand smoke either at home or at their workplace. A study among Malay housewives in Negeri Sembilan, Malaysia showed that those with low educational and socioeconomic background were more exposed to cigarette smoke inside the house rather than outside [48]. However, the same study also found that only 20% of the women were tolerant to having someone smoking near them. This might have something to do with women empowerment where women with higher education has more control over decision-making [49] and unique to Malay women in Negeri Sembilan who came from a matriarchal society (*Adat Pepatih).* In contrast to Negeri Sembilan, the Malay societies in Terengganu are patriarchal (*Adat Temenggong).* Since our study population also consists of women with lower educational background coupled with ethnic, cultural norms and religious teachings which is to honor and respect their family members especially their husbands, most women in our study might not be as empowered or felt they have the rights to tell their male family members not to smoke near them. The known negative effects of passive smoking that have been well studied should be an important enough reason to empower and encourage women that they do have the right to prevent any person smoking in close proximity to them.

Among the strengths of this study is the design (case control study) where it measures the odds of having rare disease (IHD is considered rare among women especially in east coast of Peninsular Malaysia) more efficiently. This study also explored many factors using validated questionnaire which to our knowledge has never been done before among predominantly Malay, Muslim women.

However, our study is limited by sample size, which cannot be increased due to time and logistic constraint. Due to the relatively small sample size, some factors had a very small number for example smoking status and had to be dropped from regression analysis. There is a possibility of selection bias (especially in the selection of controls); and measurement bias, which were overcome by using calibrated weighing scale and standardized questionnaire in the data collection. Smoking status and passive smoking were based on self-report and were not validated by objective measurements of biochemical markers of smoking or exposure to smoking (for example serum cotinine test).

For future research, since exposure to secondary smoke is a modifiable factor, more exploration on qualitative and quantitative research should be conducted. Information should be obtained on smoking exposure whether they were exposed mainly at their home or elsewhere and the dose of exposure to tobacco smoke. Biochemical markers for example cotinine can also be monitored as an objective measure of passive smoking. Detailed information on physical activity habits and activities may help the government prepare suitable exercise infrastructure for the population. Our research studied only the fat intake of the respondents based on the available local food. A 24-h dietary history might give a better picture of the eating habits of these women.

## Conclusion

Passive smoking has been shown to be a significant factor of developing IHD in this group of Malaysian women from an in East Coast state of Peninsular Malaysia. Other significant factors were DM, low HDL-cholesterol level and positive family history of IHD. Although all the “traditional factors” have been exhaustively studied elsewhere, there are still a lot of these factors which were not studied taking the local socio-cultural practice into considerations especially for women who in most society will be more strongly dictated by socio cultural and religious norms.

Findings from this study contribute to the knowledge on the risk factors of IHD among women especially Malay, Muslim women and this knowledge can be used for public health planning and interventions, policy making and also infrastructure implementations for prevention of IHD in similar groups.

## Abbreviations

ACS: Acute coronary syndrome
aOR: Adjusted odd ratio
BMI: Body Mass Index
CCF: Congestive cardiac failure
CI: Confidence interval
DBP: Diastolic blood pressure
DM: Diabetes mellitus
GHQ-12: General Health Questionnaire − 12
HDL-C: High density lipoprotein cholesterol
IHD: Ischemic heart disease
IPAQ-M: The International Physical Activity Questionnaire- Malay version
LDL-C: Low density lipoprotein cholesterol
NHMSIII: National Health and Morbidity Survey
NSTEMI: Non-ST elevated myocardial infarction
PS2: Power and Sample Size 2 program
RM: Ringgit Malaysia
SBP: Systolic blood pressure
SLE: Systemic lupus erythematosus
SPSS: Statiscal Package for Social Sciences
STEM1: ST elevated myocardial infarction
WC: Waist circumference
